# Improving resolution of public health surveillance for human *Salmonella enterica *serovar Typhimurium infection: 3 years of prospective multiple-locus variable-number tandem-repeat analysis (MLVA)

**DOI:** 10.1186/1471-2334-12-78

**Published:** 2012-03-31

**Authors:** Vitali Sintchenko, Qinning Wang, Peter Howard, Connie WY Ha, Katina Kardamanidis, Jennie Musto, Gwendolyn L Gilbert

**Affiliations:** 1 Centre for Infectious Diseases and Microbiology-Public Health, Institute of Clinical Pathology and Medical Research, Westmead Hospital, Sydney, NSW 2145, Australia; 2 Sydney Emerging Infections and Biosecurity Institute and Sydney Medical School-Westmead, University of Sydney, Sydney, NSW 2006, Australia; 3 Centre for Health Protection, NSW Ministry of Health, Sydney, NSW 2059, Australia

**Keywords:** Salmonella, Molecular epidemiology, Subtyping, Genotype clustering

## Abstract

**Background:**

Prospective typing of *Salmonella enterica *serovar Typhimurium (STM) by multiple-locus variable-number tandem-repeat analysis (MLVA) can assist in identifying clusters of STM cases that might otherwise have gone unrecognised, as well as sources of sporadic and outbreak cases. This paper describes the dynamics of human STM infection in a prospective study of STM MLVA typing for public health surveillance.

**Methods:**

During a three-year period between August 2007 and September 2010 all confirmed STM isolates were fingerprinted using MLVA as part of the New South Wales (NSW) state public health surveillance program.

**Results:**

A total of 4,920 STM isolates were typed and a subset of 4,377 human isolates was included in the analysis. The STM spectrum was dominated by a small number of phage types, including DT170 (44.6% of all isolates), DT135 (13.9%), DT9 (10.8%), DT44 (4.5%) and DT126 (4.5%). There was a difference in the discriminatory power of MLVA types within endemic phage types: Simpson's index of diversity ranged from 0.109 and 0.113 for DTs 9 and 135 to 0.172 and 0.269 for DTs 170 and 44, respectively. 66 distinct STM clusters were observed ranging in size from 5 to 180 cases and in duration from 4 weeks to 25 weeks. 43 clusters had novel MLVA types and 23 represented recurrences of previously recorded MLVA types. The diversity of the STM population remained relatively constant over time. The gradual increase in the number of STM cases during the study was not related to significant changes in the number of clusters or their size. 667 different MLVA types or patterns were observed.

**Conclusions:**

Prospective MLVA typing of STM allows the detection of community outbreaks and demonstrates the sustained level of STM diversity that accompanies the increasing incidence of human STM infections. The monitoring of novel and persistent MLVA types offers a new benchmark for STM surveillance.

A part of this study was presented at the MEEGID × (Molecular Epidemiology and Evolutionary Genetics of Infectious Diseases) Conference, 3-5 November 2010, Amsterdam, The Netherlands

## Background

Food-borne diseases are responsible for considerable morbidity, mortality and economic cost [1-3]. The global human health impact of non-typhoidal *Salmonella *infection can be as high as 93.8 million illnesses and 155,000 deaths each year [1]. Many cases of salmonellosis could be prevented if common outbreak sources could be identified rapidly, thus enabling earlier public health interventions. However, increased mobility of people and complexity of food production, processing and distribution systems have complicated the recognition and investigation of outbreaks [4]. Salmonella-related outbreaks are increasingly associated with a diverse range of sources, yet the mechanisms of contamination often remain poorly understood [4,5]. Purely epidemiological approaches to distinguishing outbreaks from sporadic cases significantly underestimate the proportion of outbreak cases. Furthermore, linking laboratory results to public health actions and increasing the timeliness of case follow-up are critical to reducing delays in the investigation of outbreaks [6]. Recent evidence suggests that the geospatial clustering of food-borne isolates supports a more timely detection of otherwise unrecognized outbreaks [7].

*Salmonella enterica *subsp. *enterica *serovar Typhimurium (*S*. Typhimurium/STM) has been predominant for decades in Australia and elsewhere in the world [8]. It represents approximately one third of human and one quarter of bovine and chicken isolates, albeit with considerable geographic and temporal variation. Multilocus sequence typing of STM isolates from sub-Saharan Africa suggests the continuous evolution of STM towards a more human-adapted invasive life-style, similar to that seen in *S*. Typhi [9]. STM is a diverse serovar and additional subtyping is needed for outbreak detection and investigation. Traditionally, the method of choice has been phage typing, which is based on the susceptibility of isolates to a panel of 34 bacteriophages. The current phage typing scheme recognises 207 definitive and many more provisional phage types [10]. Over the past 5 years, phage types 9, 170, 135 and 135a accounted for 70% of the STM isolates received by the Enteric Reference Laboratory in New South Wales. Clusters are difficult to recognise among common phage types and a more discriminatory subtyping method is needed. Numerous subtyping methods for STM have been used previously, including pulsed-field gel electrophoresis (PFGE), IS*200 *restriction fragment length polymorphism (RFLP) and amplified fragment length polymorphism (AFLP), but all have disadvantages [11-13]. Recently, multiple locus variable-number tandem-repeat (VNTR) analysis (MLVA) has been suggested as a rapid alternative to RFLP [14,15], capable of discriminating strains within the most common phage types [14].

While typing methods have usually been used to confirm epidemiological links, the rapid turn-around time of PCR-based methods makes them suitable for the prospective detection of clusters for further investigations [16]. Other advantages of MLVA are the relative ease of implementation and harmonisation of the method between laboratories [17]. The emerging evidence suggests that MLVA is superior to RFLP and PFGE for both surveillance and outbreak investigations [15] and that prospective monitoring of the local epidemiology of STM using MLVA can be beneficial for tracing possible sources of community cases of salmonellosis [18-21]. However, these studies were based on relatively small numbers of isolates and did not detail specific insights that such high resolution typing could provide.

In our previous study [22], we described the importance of scalable cluster definitions adjustable to reflect changes in local STM disease prevalence and the availability of public health resources. The objectives of this project were to explore the MLVA dynamics of human STM infection in a 3-year prospective study of STM MLVA typing for public health surveillance. We also tested the sensitivity of STM MLVA cluster definition in a relatively low prevalence country, using New South Wales (NSW), the most populous state of Australia, as an example.

## Methods

### Bacterial strains

All isolates in this study had been referred to the NSW State Enteric Reference Laboratory at the Centre for Infectious Diseases and Microbiology, Institute of Clinical Pathology and Medical Research (ICPMR) in Sydney, Australia. All isolates presumptively identified as *Salmonella *species by pathology service providers in NSW are referred for confirmatory testing and serotyping, according to the Kauffmann-White-Le Minor scheme [10] and subsequent phage typing [23]. The latter is conducted by Microbiological Diagnostic Unit (MDU, University of Melbourne). During a 3-year period between August 2007 and September 2010, all confirmed STM isolates were also fingerprinted using MLVA as part of the NSW public health surveillance program. STM isolates were batched and tested weekly. The food isolates were from various sources collected by the Division of Analytical Laboratories, ICPMR, Sydney, Australia. Isolates were grown overnight on blood agar plates, and a small loopful of colonies was used for DNA extraction by boiling for 10 min. The neat supernatant was used as a source of DNA templates for PCR amplification.

### PCR amplification

The multiplex MLVA PCR reactions to detect five variable number tandem repeats (STTR9, STTR5, STTR6, STTR10pl and STTR3) were performed as described previously [14,24]. Briefly, amplifications of five loci were achieved in 30 μL reactions prepared using Qiagen HotStar *Taq *PCR Mix (Qiagen, Germany) and a primer concentration of 0.1-0.2 μM. The forward primers for loci STTR3, STTR5, STTR6, STTR9 and STTR10pl were labelled with fluorescent dyes FAM, NED, FAM, HEX and HEX, respectively. The amplification conditions were as follows: an initial denaturation step at 95°C for 15 min, followed by 25 cycles of 94°C for 30 s, 60°C for 1.5 min, and 72°C for 1.5 min, with a final extension at 72°C for 10 min. Standard gel electrophoresis was used for visualisation of amplification products if required. A single target PCR was run for any isolates with missing PRC amplicons, to confirm absence of amplification.

### Fragment and sequence analysis

PCR products were analysed on an ABI 3130xl Genetic Analyzer (Applied Biosystems). The size and dye label associated with each amplicon were determined using Peak Scanner software v.1.0 (Applied Biosystems). Initially, a set of calibration STM strains with VNTR loci of different sizes were initially sequenced to verify the method and to normalise the raw data to be obtained from prospective MLVA typing. Sequence analysis was performed using an ABI 3130xl Genetic Analyzer (Applied Biosystems). The sequencing primers were non-labelled MLVA amplification primers. PCR product sizes from these strains were plotted against the observed number of repeats for each locus and these plots were used for the determination of the number of repeat units in each locus (Additional file 1: Figure S1). This information, along with the size of the flanking region (i.e., the size of the amplicon for a given locus excluding any repeat sequences) [25], was employed to determine the number of repeat sequences present and to assign an allele number [24]. All MLVA results were reported as a string of five numbers (STTR9-STTR5-STTR6-STTR10pl-STTR3), as described previously [24], where the first four digits represent numbers of repeats and the fifth the total length of the sequence at the corresponding loci. At each locus, alleles or fragments were assigned into 'size bins', ensuring that minor size variations in PCR fragment analysis were given the same allele number [14]. If no amplicon was generated, the allele '0' was assigned. A strain of *S*. Typhimurium LT2 (complete genome sequence available from GenBank Accession Number NC_003197) was used as a positive control during the analysis and gave the expected allele profile of 5-15-14-11-490.

### Clustering of isolates and epidemiological analysis

A cluster was defined as five or more isolates with the same MLVA type collected over a period of 4 weeks [15]. Cluster case density was defined as the number of days from the specimen collection date of the first cluster isolate to the collection date of the third isolate and was categorised into cluster case densities of 1-7, 8-14 and > 14 days coded as density 1, 2 and 3, respectively [26]. Spatial cluster density 1 was assigned to clusters with over 50% of cases located in neighbouring postcodes. Spatial cluster densities 2 and 3 were assigned to clusters with 10-50% of cases located in neighbouring postcodes and clusters with a random spatial distribution of cases, respectively. Automated searches were conducted weekly through the database containing STM MLVA patterns, dates of specimen collection and postcodes of patients' residence. The clusters were spatially visualised using the biosurveillance portal of the Centre for Health Informatics, University of NSW (http://www2.chi.unsw.edu.au/biosurveillance/index.php). Clusters with spatial density 1 and 2 were flagged for public health assessment.

### Statistical analysis

Descriptive statistics and regression analyses were performed. The relative confidence intervals at 95% were calculated and a value of *P *< 0.05 was considered significant for all the tests. The sensitivity of our cluster definition was tested using linear regression. Simpson's index of diversity (DI) was calculated to assess the discriminatory power of MLVA types [27]. The richness of STM populations was estimated by McIntosh's dominance index:

$$D=\frac{\text{N}-\surd \sum {{\text{n}}^{2}}_{\text{i}}}{\text{N}-\surd \text{N}}$$

where *D *is McIntosh's dominance index of diversity, *n_i _*- the proportional abundance of the *i*th MLVA types, and N is the total number of STM isolates in a given population [28].

## Results

### Discriminatory power of MLVA typing of *Salmonella *Typhimurium isolates

A total of 4,920 STM isolates were genotyped using the MLVA method. Following the removal of duplicate isolates from the same episode of the disease, a subset of 4,377 human isolates was included in the analysis (Table 1). The STM spectrum was dominated by a small number of phage types, including DT170 (44.6% of all isolates), DT135 (13.9%), DT9 (10.8%), DT44 (4.5%) and DT126 (4.5%). The set of 607 isolates of DT135 included 325 isolates designated to phage type 135a variant originally described by Australian Salmonella Reference Centre at IMVS, Adelaide (53.5%).

**Table 1:** Distribution of phage and MLVA types for all isolates with a phage type abundance > 1% of the total number of isolates

Most common phage types	No. isolates (% of total)	No. MLVA types	No. isolates with most common MLVA type (%)
170	1,953 (44.6)	112	1536 (78.6)

135 (including 135a)	607 (13.9)	151	329 (54.2)

9	473 (10.8)	113	315 (66.6)

RDNC	206 (4.7)	69	17 (8.2)

44	197 (4.5)	14	87 (44.1)

126	197 (4.5)	33	61 (30.9)

193	101 (2.3)	18	27 (26.7)

6	95 (2.1)	32	17 (17.9)

197	69 (1.6)	27	31 (44.9)

2	62 (1.4)	14	15 (24.2)

29	59 (1.3)	8	43 (72.9)

Not typable	48 (1.1)	28	5 (10.4)

Others	310 (7.1)	-	-

All isolates	4,377 (100)	667	-

Notes: MLVA, multi-locus variable number tandem repeat analysis; RDNC (Reacts but Does Not Conform)

**Figure 1:**
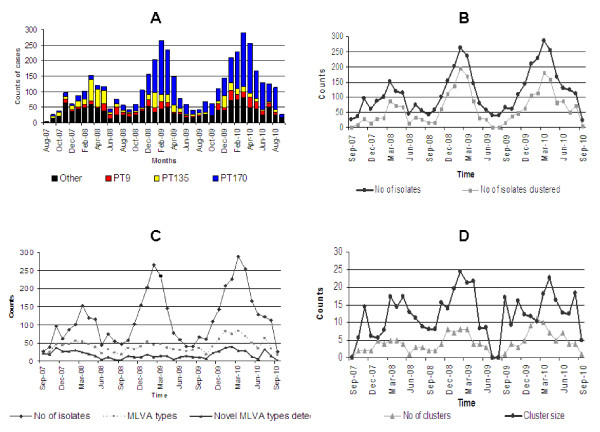
**Trends of STM phage types and MLVA patterns over the 3-year study period**. **A: **Monthly counts of STM phage types. Cumulative counts for phage types DT170, DT135 and DT9 are colour-coded. All other phage types are included in 'other' types. **B**: Monthly occurrence of total MLVA types and novel MLVA types observed. **C**: Monthly counts of STM isolates included in MLVA clusters. **D**: Monthly counts of MLVA clusters and trends in MLVA cluster sizes.

There was a continuous increase in the number of STM infections during the study and the relative contributions of common phage types to local STM activity changed over three seasonal peaks. DT135 predominated in 2008, but was subsequently replaced by DT170 as the predominant phage type in 2009 and 2010, with a concomitant increase in the activity of DT9 (Figure 1-A). In total, 667 different MLVA types or patterns were observed. Three related MLVA patterns (3-9-7-12-523, 3-9-7-13-523 and 3-9-8-12-523) belonging to DT170 included almost one third (30%) of all STM isolates.

**Figure 2:**
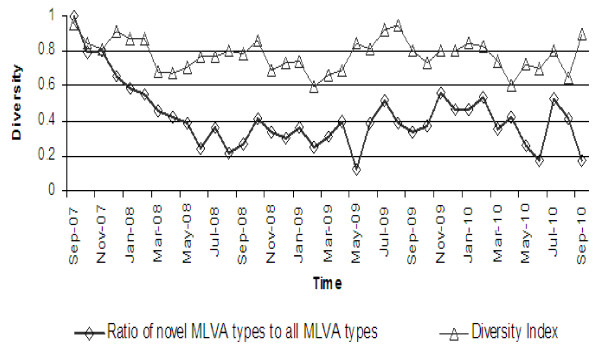
**Temporal changes in STM population diversity represented by monthly figures of McIntosh's dominance index of diversity and the ratio of novel MLVA types to all MLVA types observed within a given month of the study**.

The greatest number of MLVA types were recorded in DT135 (151 in total, including 87 types among phage type 135a isolates) and DT9 (113). In contrast, the dominant STM DT170 demonstrated only 112 MLVA types in the set of 1,953 isolates. One MLVA type represented 78.6% of the DT170 set while only 66.6%, 54.2%, and 44.1% of phage types DT9, DT135, and DT44 consisted of a single MLVA type, respectively (Table 1). There were differences in the discriminatory power of MLVA typing within endemic phage types: Simpson's index of diversity ranged from 0.109 and 0.113 for DT9 and DT135 to 0.172 and 0.269 for DT170 and DT44, respectively (Figure 2).

### Diversity and temporal dynamics of MLVA patters

Sixty-six distinct STM clusters were observed in this study. They ranged in size from 5 to 180 cases and in duration from 4 to 25 weeks. Endemic MLVA patterns 3-9-7-12-523, 3-9-7-13-523 and 3-9-8-12-523 belonging to DT170 were responsible for 11 clusters lasting for three or more months in 2009-2010. Forty-three clusters were due to novel MLVA types and 23 represented reappearance of previously recorded MLVA types. The increase of STM cases during summer months (December-February in the Southern Hemisphere) was reflected in increases in the number of cases of salmonellosis and MLVA types detected in the community (Figure 1-B). The proportions of STM isolates included in clusters also showed seasonal fluctuations corresponding to changes in STM incidence (Figure 1-C). There was no significant change in the number of clusters or their average size over the period of this study (Figure 1-D).

At the beginning of the study all, and then the majority of, MLVA types were considered to be 'novel' as they had been registered for the first time. The ratio of novel MLVA types to all MLVA types observed stabilised in the five months after the start of prospective surveillance. After that, between 20% and 60% of all MLVA types detected in the study at any time consisted of novel, previously unreported types. The diversity of the STM population remained relatively constant over time with McIntosh's dominance index of diversity fluctuating between 0.6 and 0.9 (Figure 2). A subset analysis of 15 groups of recurrent STM MLVA clusters (consisting of 1-2 recurrences each) revealed differences in size, duration and spatial density between initial clusters (15 clusters) and corresponding reoccurred clusters (24 clusters) due to the same MLVA type (Table 2). The latter were larger than initial clusters (66 cases per cluster on average versus 23 cases, respectively, *P *= 0.001) and less spatially dense (overall spatial density 2.75 versus 1.95, respectively, *P *< 0.001). Differences in temporal duration and density were not statistically significant (Table 2). Over the 3-year study period, there was no significant change in the number of MLVA clusters or proportion of STM isolates clustered. There was a non-significant trend towards a decrease in the size of individual clusters (Figure 2).

**Table 2:** Differences between primary and re-emerged clusters of STM

	Primary^1 ^clusters (n = 15)		Re-occurred^2 ^clusters (n = 24)		Statistical significance
	
	Mean	CI (95%)	Mean	CI (95%)	
Cluster size (number of cases)	22.66	12.78	66.46	42.18	*P *= 0.005

Duration (weeks)	7.21	3.98	12.04	0.27	*P *= 0.07

Temporal density	1.4	3.95	1.47	0.27	NS

Spatial density	1.95	0.49	2.75	0.18	*P *< 0.001

Notes: ^1 ^A primary cluster is the first due to a new MLVA type

^2 ^A re-occurred cluster is a second or subsequent cluster due to the same MLVA type as the corresponding primary cluster

### MLVA clustering and public health follow-up

**Figure 3:**
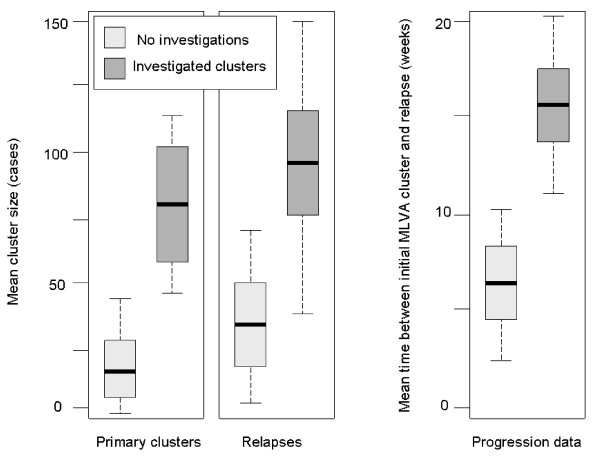
**Box plots of the mean MLVA cluster sizes and time between an initial cluster and its re-occurrence**. Mean value is shown as horizontal line across the bar. Ends of a bar designate confidence intervals and dotted lines indicate the spread of values in the subgroup.

Twenty four MLVA clusters were investigated during the study. Fifteen clusters (15/66 or 22%) were subsequently investigated by public health authorities and had epidemiological links confirmed and the likely source established by environmental testing (Table 3). Of note, epidemiological investigations of four of those clusters were initiated as a result of alerts from prospective laboratory surveillance. The majority of these outbreaks were linked to eateries or food shops and contaminated eggs, chicken or pork meat were implicated as potential sources of infection. Investigations of nine clusters failed to identify a common source, often due to poor food recall. Despite public health interventions resulting in the elimination of STM contamination in food outlets, five of the 15 (33%) STM clusters of the same MLVA type re-occurred during the period of the study. The re-occurrence rate for clusters that did not trigger any public health actions was similar (17/50; 34%). However, clusters of the same MLVA type following initial public health interventions re-occurred on average 9 weeks later than those that had not been followed up epidemiologically (16.3 weeks between clusters versus 7.5 weeks, *P *< 0.001, Figure 3).

**Table 3:** Clusters identified by MLVA typing with confirmed epidemiological links

Cluster Number	Time	**MLVA type **[24]** (Phage type)	Sources of infection identified by epidemiological investigations
1	Oct 2007	4-16-13-0-517 (DT170)	Contaminated chicken meat from a sushi shop

2	Oct-Nov 2007	3-10-8-9-523 (DT44)	Use of raw eggs (cheesecake) at a common bakery, interstate clusters in NSW, Victoria and South Australia

3*	Nov 2007	3-11-10-8-523 (DTU302, U290)	Raw egg milkshake consumed by three siblings

7	Nov 2007	3-20-17-11-523 (DT9)	Meal prepared and cooked at home function (deep-fried ice cream)

4	Jan-March 2008	3-9-8-12-523 (DT170)	Raw eggs in custard for a cake at private party consumed by some cases

5	Feb-March 2008	3-17-16-13-523 (DT126)	Linked to local egg supplier, confirmed by environmental sampling

6	May 2008	3-12-10-12-523 (U290)	Restaurant outbreak, environmental samples from restaurant confirmed the link.

8	Sept-Oct 2008	3-10-14-11-496 (DT9)	Linked to eating mousse made with raw eggs

9	Nov 2008	3-9-7-13-523 (DT170)	Interviews indicated restaurant outbreak. Cross- contamination of pesto suspected.

10	Nov 2008	3-17-16-13-523 (DT126)	Linked to raw eggs contamination

11	March 2009	3-9-7-12-523 (DT170)	Fried ice cream positive and raw beef positive. Chopping board swabs and salad samples were positive for STM. One point source outbreak in the middle of endemic activity of PT170

12*	Feb-April 2009	3-9-8-12-523 (DT170)	Restaurant related outbreak

13*	March-April 2010	3-9-7-12-523 (DT170)	Restaurant related outbreak. Epi link confirmed by positive cultures from the chef and his family.

14	June 2010	3-9-7-14-523 (16 cases) 3-9-7-15-523 (15 cases) (DT170)	Cases from narrow geographic area. Interviews indicated kebab store outbreak, later confirmed by detection of undistinguishable STM MLVA type in chicken, tabouli and hummus.

15*	Aug 2010	3-9-7-13-523 (DT170)	Human cases from narrow geographic area. Epidemiological link to deep-fried ice cream made with raw eggs. Farm inspection not conducted.

* Public health investigation was initiated by STM MLVA cluster alerts

** The STTR3 fragment size of the last allele in MLVA profile can be translated into the number-of-repeat-nomenclature [25], i.e. 496 = 0112, 517 = 0311, 523 = 0212

### Analysis of MLVA cluster definition

**Figure 4:**
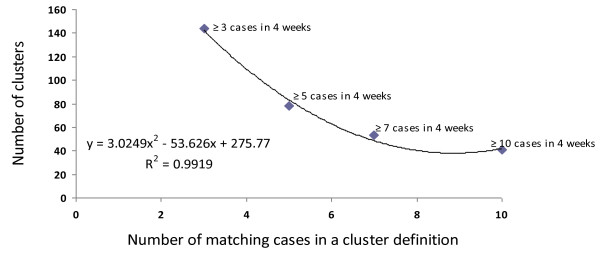
**Sensitivity of cluster definition**.

The sensitivity of a STM MLVA typing based cluster definition was tested to identify the association between the number of cases (x) with matching STM MLVA types per 4-week period and the number of clusters (y) potentially reported. Four cluster definitions (Figure 4) were contrasted, including (i) three or more cases; (ii) five or more cases; (iii) seven or more cases, and (iv) ten or more cases of matching MLVA type obtained in a 4-week time period. The regression model expressed the relationship: y = 3.025 × ^2 ^- 53.63 × + 275.8. Sensitivity analysis of MLVA typing-based clustering identified 33-91 MLVA clusters based on different definitions, involving 77-83% of all STM isolates tested. The decrease of the number of cases to three or more would significantly increase the number of clusters reported. The average number of isolates per cluster varied between 8.6 (range 2-81) and 19.3 (range 5-166). The change in the minimum duration of a cluster from 2 to 8 weeks did not significantly affect the final number of MLVA clusters detected.

## Discussion

Our findings extend previous studies by providing additional evidence on the utility of the prospective MLVA typing of STM. Prospective MLVA typing of STM improves the resolution of outbreak detection, breaks down each phage type into several MLVA profiles and offers new insights into the diversity of genotypes. At the same time, our results emphasize important variations in the discriminatory power of MLVA typing. We confirmed the previous observation [20], based on a set with different distribution of phage types (DT101, 104 and 160) [20], that the locus STTR10pl has the greatest number of different alleles, followed by STTR5 and STTR6.

The role of appropriate cluster definition was one of the most important lessons learnt from our study. Our regression analysis confirmed the validity of the initial cluster definition that was suggested for low prevalence settings [15] and quantified the impact of increasing either the total number of cases or duration of a cluster (or both) on the sensitivity of its definition (Figure 4). The choice of cluster definition is heavily influenced by the local prevalence of infection and public health capacity to respond to surveillance alerts [29] so the definition should be optimised for the local prevalence and distribution of STM MLVA patterns.

Our findings add further insights into the epidemiology of sporadic STM infection and offer a new way to document baseline STM disease burden. The relative stability of the ratio of novel MLVA types to all MLVA types observed at any given time suggests constant seeding from new or established STM infections and environmental sources. The monitoring of this ratio together with established measures, such as McIntosh's dominance index of diversity, could provide a useful benchmark for STM surveillance. Unfortunately, we did not observe any measurable impact of prospective MLVA typing on the trends of STM infections, perhaps due to the relatively short duration of the study and to the limited number of clusters that were investigated by public health units. A significant proportion of STM MLVA clusters had no obvious temporal and spatial clustering and was associated with endemic MLVA types. This is not surprising, as *Salmonella *infections have been linked to a range of food vehicles including eggs, chicken, beef, pork, dairy products and salad vegetables [30,31] with an attack rate of 7-10% [32], making public health control of human salmonellosis particularly challenging. The continuous introduction of new STM variants and re-introduction of endemic variants from different food outlets and farms offered an additional insight into the inherent complexity of controlling the STM endemicity and the importance of addressing the whole food chain [31]. As in previous reports [31], our STM MLVA typing identified identical patterns in isolates from the same source and from different sources at different points in time, demonstrated co-circulation and persistence of endemic STM types and occasionally found similar MLVA profiles in epidemiologically unlinked cases.

To our knowledge, this is the first report of the association between STM MLVA-guided public health interventions and the diversity of STM infections in a community. We did not detect any significant impact of MLVA-based surveillance on the size, number or the duration of human STM infections. The apparent delay in the re-occurrence of MLVA types linked to public health interventions is of particular interest. However, these observations and their possible cause-effect relationships require further confirmation in a larger longitudinal study with more detailed epidemiological data collection to enable the development of quantitative rules to exclude random effects and to differentiate primary clusters from secondary re-occurrences in low-endemicity settings.

Several potential limitations of this study should be acknowledged. First, the study was limited to one state of a large country with a disproportionate concentration of STM cases in one metropolis (i.e. Sydney) and lacked sufficient numbers of isolates with diverse MLVA types required to perform statistical analyses of causal associations. A study with statistical power for these analyses would require a decade of data collection, as multi-centre assessment may not be achievable due to potential disparities in local STM epidemiology and public health capacity. In addition, a limited number of endemic MLVA patterns constituted almost a third of all STM isolates. However, the epidemiology of STM infection and public health responses in New South Wales are similar to those in other regions of Australia and Western Europe [33,34], supporting the generalisability of our findings. Second, we relied on a single method of typing due to the logistics of our rapid prospective public health surveillance and restricted our cluster definition to STM isolates with identical MLVA patterns and excluded isolates differing by one repeat in one of five loci [20]. Occasionally, such closely related isolates might represent epidemiologically linked cases of infection and alternative typing methods might be useful to clarify the relationships between those strains. However, it is unlikely that our more restrictive case definition would significantly affect the clustering rates or our overall conclusions. Third, we assumed the stability of the MLVA loci over the period of the study. While existing evidence supports this assumption [35], the dynamics of MLVA loci over an extended period of time deserve further evaluation. Furthermore, minor variations in laboratory protocols may complicate the inter-laboratory comparison of MLVA results, therefore efforts to harmonise STM MLVA testing and to develop a unified nomenclature have received well-deserved attention [25,17]. Lastly, the trends observed in the study apply to a scenario of relatively low incidence of food-borne STM infections with access to laboratory testing and mandatory STM notification. They may not be readily generalizable to high STM endemicity settings with larger population densities and different diagnostic or public health practices.

## Conclusions

Prospective genotyping of STM by MLVA can be used for the public health surveillance of sporadic and outbreak cases and in tracing possible sources of community outbreaks. The definition of STM MLVA clusters with sufficient sensitivity for timely interventions could be optimised for the local prevalence of infection and distribution of STM MLVA patterns. The study demonstrated the sustained levels of diversity of STM types that accompany an increasing incidence of human STM infections. Monitoring the STM population diversity can provide a useful benchmark for STM surveillance and an additional indicator for the assessment of possible impacts of public health interventions.

## Competing interests

The authors declare that they have no competing interests.

## Authors' contributions

VS conceived the study, undertook analysis and drafted the manuscript. QW and GLG initiated the study and made major contribution to the study design. PH, CWYH, JM and KK worked on the collection of data used in the manuscript and drafted the manuscript All authors contributed to the writing of the manuscript and read and approved the final manuscript.

## Pre-publication history

The pre-publication history for this paper can be accessed here:

http://www.biomedcentral.com/1471-2334/12/78/prepub

## Supplementary Material

Additional file 1 **Figure S1 Plots of observed number of repeats for each locus against sizes of sequenced VNTR regions**. Click here for file
