# Supplementary material for: Improving resolution of public health surveillance for human Salmonella enterica serovar Typhimurium infection: 3 years of prospective multiple-locus variable-number tandem-repeat analysis (MLVA)
Source: BMC Infect Dis. 2012 Mar 31;12:78. doi: 10.1186/1471-2334-12-78 (PMC3368731; doi:10.1186/1471-2334-12-78)
Supplement: Additional file 1 — Figure S1 Plots of observed number of repeats for each locus against sizes of sequenced VNTR regions. [file 1471-2334-12-78-S1.PDF]

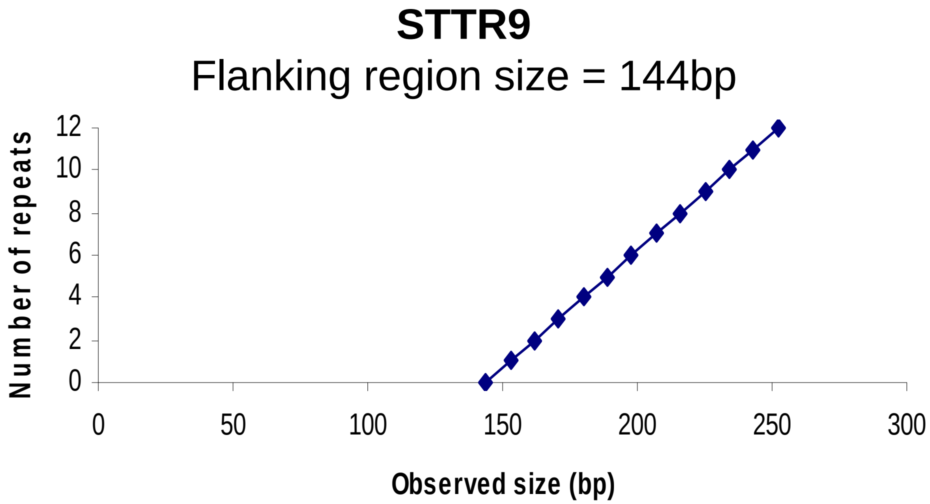
STTR9
Flanking region size = 144bp
Number of repeats
0
2
4
6
8
10
12
0
50
100
150
200
250
300
Observed size (bp)

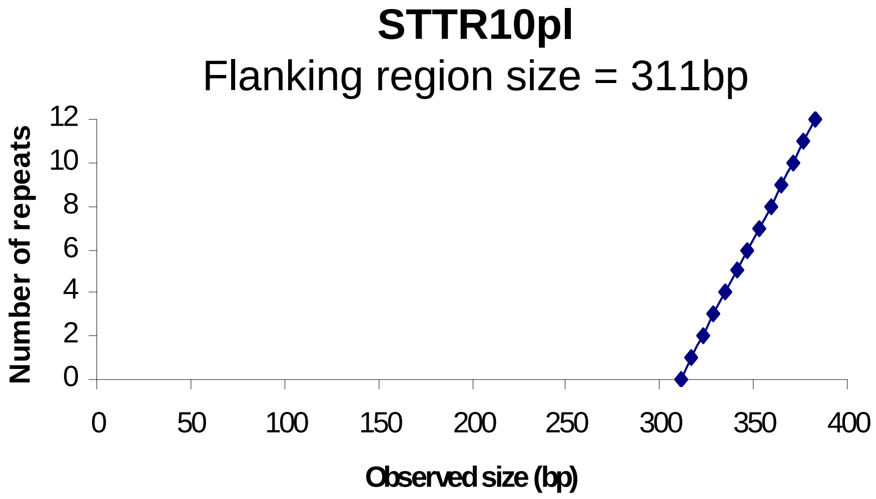
STTR10pl
Flanking region size = 311bp
Number of repeats
0
2
4
6
8
10
12
0
50
100
150
200
250
300
350
400
Observed size (bp)

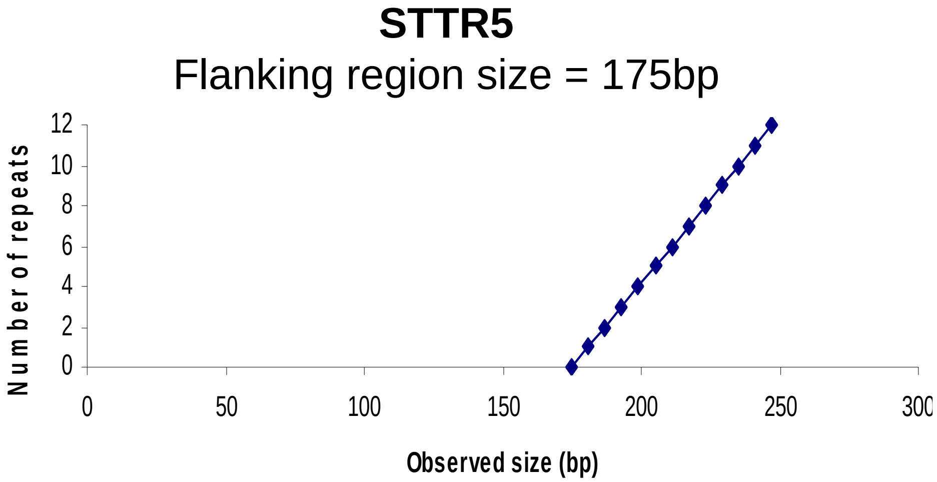
STTR5
Flanking region size = 175bp
Number of repeats
0
2
4
6
8
10
12
0
50
100
150
200
250
300
Observed size (bp)

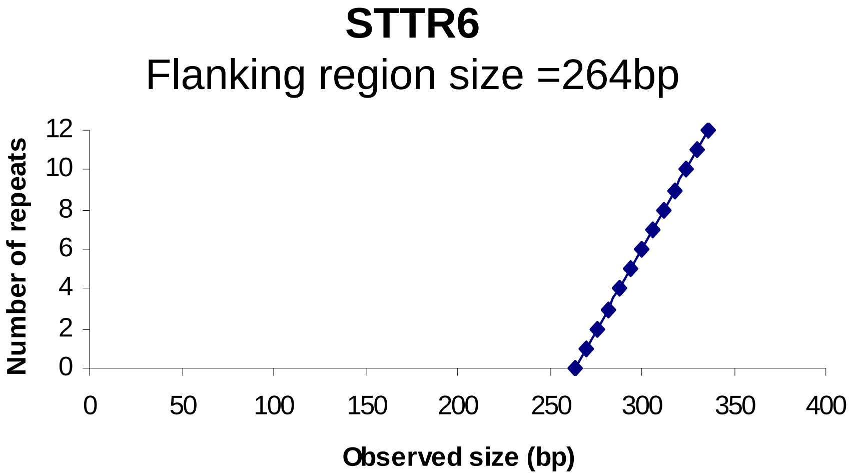
STTR6
Flanking region size =264bp
Number of repeats
0
2
4
6
8
10
12
0
50
100
150
200
250
300
350
400
Observed size (bp)
